# The Hospitals of Insulinde

**Published:** 1912-08-03

**Authors:** 


### THE HOSPITALS OF INSULINDE.

### Their Construction, Administration, and Mortality.

(BY OUR SPECIAL COMMISSIONER.)

A recent trip to the Dutch East Indies—better called Insulinde, that being the general term given to these colonies by Douwes Dekker in 1860—enabled me to make some notes on the various hospitals which are to be found there. It must be stated that while the majority of these are of the type known as "temporary hospitals" they compare more than favourably with similar institutions I previously inspected in the West Indies and the Southern States of North America. These temporary hospitals are temporary only in the sense that the older sheds of the Moabite at Berlin are temporary. They are all built on the pavilion system, the pavilions being always one-storeyed, and the floor of the wards being generally raised only a few inches above the ground. Exceptions are found in Sumatra, where for various reasons the buildings are on short piles, allowing free ventilation below. This, of course, necessitates the patients being carried in on specially constructed stretchers, but it has several compensating advantages. The structure of the walls is generally wood, sometimes lined with brick. The roofs are of hardwood tiles, with sloping eaves. In such a humid climate as Insulinde possesses, hospital construction offers various problems which have not yet been satisfactorily solved. One of the most difficult is that of efficient wall drainage, which is generally bad. In districts where it rains almost every day, it is extremely difficult to keep the wards damp-free, and usually it is quite impossible to come even near the ideal.

### The Nursing Home at Soerabaya.

A word must be said about the private nursing homes. There are several of these, but perhaps the best of all is the fine nursing home at Soerabaya. This is established in a large stone building, the front villa being used as the administrative block, while the surrounding blocks are used for the accommodation of the patients. Each patient has a large lofty room, admirably but plainly furnished. The floors are of marble, or of ordinary red tiles, generally relieved by a few carpets. Each bed is furnished with the regulation *klamboe* or mosquito-net, which is now also found in the soldiers' barracks. This huge tent-like covering naturally interferes somewhat with the proper nursing of the patients, but is absolutely indispensable if the occupant of the bed is to be at peace and undisturbed by the obnoxious little insects, against which the fumes of petroleum and *seri* or citronella oil do not seem to guard the European. There is an excellent up-to-date operating table, and adequate provision is made for *x*-ray diagnosis, serum and bacteriological investigation, and for modern methods of treatment.

### An Elaborate System for Private Patients.

The system followed in this home, and generally in Insulinde in the case of private patients, differs considerably from that which obtains with us. For instance, the patient who comes in to have an operation for appendicitis performed is asked who is his chemist. When he comes to pay his fee, he is presented with a detailed account setting forth every item that has been used during the treatment. He is asked to pay for the dressings so much, for the medicines supplied so much, for the instruments required so much, in case special appliances have been found necessary, and in fact for everything that has been used during the operation. A charge is made for the use of the room and for the food and nursing, but alcoholic drinks and comforts are specially enumerated.

The system has its obvious advantages, for it shows the patient clearly what has been required and makes him fully sensible of the gravity of the operation. The surgeon's, anæsthetist's, and assistants' fees are matters for private settlement, and it may be said that the fee for a major operation here compares favourably, from the patient's point of view at least, with that charged in England. The scale is a little higher than that which prevails on the Continent, but considering the general excellence of the results, and the undoubtedly expert technique used, it cannot be said to be an expensive scale. The nursing is on the whole excellent. The nurses are European sisters who have been well trained in European hospitals and training schools, and their methods are up to date, while their skill is evident to anyone who witnesses the way in which they handle their charges. I was most favourably impressed with the general excellence, strict cleanliness, and admirable system that prevail at this fine institution, of which Soerabaya has reason to be proud.

### The Buitenzorg Mental Hospital.

At Buitenzorg there is a large Government mental hospital for both native and European patients. The natives live very much under the same conditions of life that they possess at their *kampongs* or villages. Each patient is given a small plot of ground for tillage, the product of which he sells to the administration. The percentage of cures obtained here is relatively high, and this good result is undoubtedly due to the excellent methods of treatment. Insanity is not more common here than in Europe, but the etiological factors can hardly be said to be similar to those in England. Alcoholism and religious stress, for example, account for few cases, and these chiefly among the Europeans. Among the natives syphilis and hereditary predisposition are perhaps the main causative factors.

Here also is a hospital for the blind, which is maintained by the Government. Natives are trained as oculists, and much has been done to cope with the number of cases of preventable blindness which are so common in the East. At Batavia there is a special school for native doctors, who practise in the *kampongs* and do good work. It is also proposed to establish a school for midwives. This is greatly needed, for it is said that annually nearly fifty thousand mothers and children die as the result of the primitive methods of conducting labour which native custom has sanctioned. The most common cause of death is sepsis, and sepsis of a particularly bad kind, since it has been found that numerous cases of tetanus occur owing to infection of the navel cord when it is cut.

### Civil *versus* Military Hospitals.

In general the civil hospitals are poor, and, with few exceptions, do not impress the visitor. Mr. Kol, in his book on Java, gives a description of the horrible state in which he found one civilian hospital in an up-country town, a description which strongly reminds the reader of the account that Howard gave of some of the hospitals in his day. This hospital is now in a much better condition, but still a great deal can and must be done before the civil hospitals, as a class, can compare, even in essentials, with similar institutions in Europe.

The military hospitals, on the other hand, are almost without exception good. At Batavia, Semarang, Soerabaya, Buitenzorg, Padang, Cheribon, and Tjilitjap there are excellent military institutions which do not need to fear comparison with other hospitals of a similar nature anywhere. The fine institution at Buitenzorg is especially worth a visit. Here a separate quarter is assigned for the beri-beri patients, most of whom are recruited from Atjeh and are sent here for treatment.

### Diseases Treated.

The upland air is very suitable for the treatment of the paralysis that is a feature in this disease, and the patients recover rapidly for the most part. When the cardiac complications are grave recovery is usually doubtful, but the wonderful results obtained in the treatment of the paralysis are striking to one who sees the patients when they first come and observes them a month or two later when they are ready to return to work.

These hospitals are provided with good dispensaries, where the drugs are carefully tested, so that the patients are sure of obtaining what is prescribed—a matter of considerable importance in most military hospitals, where the delivery of the drugs is generally in the hands of a contractor, who cannot always be depended upon to give pure and unadulterated samples. Syphilis is prevalent, and the ordinary mercurial treatment is most in favour, although considerable progress has been made in the newer methods of treatment with arsenic compounds, such as salvarsan. The arrangements for quarantine and isolation are excellent. They need to be, for infectious diseases are unfortunately very common. While scarlet fever is unknown, diphtheria, plague, smallpox, cholera, dengue, and especially dysentery, are matters of ordinary and almost daily occurrence in Java. In Sumatra plague is very rare, but the other diseases all occur and must be guarded against. The Government sanitary department works well, and when a case of plague is reported prompt arrangements are made to remove the sufferer to the plague hospital, and the contacts to the isolation quarter, while the house is closed and, with its neighbours, subjected to a thorough disinfection.

### Native Vaccinators.

During my visit to Soerabaya there were several cases of plague in the town, and a small epidemic of smallpox had broken out which accounted for some forty deaths per day. Native vaccinators are employed by the Government, and every man, woman, and child in the *kampongs* is supposed to be properly vaccinated. The operation is, however, often done in a perfunctory manner, and the results cannot be depended upon. Very rightly, therefore, the Government lays great stress on proper sanitation and cleansing of the native and Chinese camps, and the visitor is struck from the first with the almost universal absence of refuse and dirt in these quarters.

### Nursing.

The question of nursing is an important one. At present few female nurses are employed. In the military hospitals, hospital orderlies act as nurses, usually very capably, but one necessarily misses the skilled attention of the trained nurse which is so marked a feature in European hospitals. Notwithstanding this lack, the results obtained in these institutions compare excellently, so far as statistics of recovery, length of stay, mortality, and daily averages are concerned, with those obtained with us. To a certain extent these good results are due to the local conditions of climate and environment, but credit must be given to the good treatment and the really excellent administration. The lavatory accommodation in most of the hospitals is fair; in many it is excellent, regard being had to the fact that many natives do not know how to use what is provided for them. In fact, a comparison with West Indian hospitals on this point shows that the Insulindian institutions are far better than those in the islands of the Caribbean Sea.

